# Comparative Analysis of Fruit Metabolites and Pungency Candidate Genes Expression between Bhut Jolokia and Other *Capsicum* Species

**DOI:** 10.1371/journal.pone.0167791

**Published:** 2016-12-09

**Authors:** Sarpras M, Rashmi Gaur, Vineet Sharma, Sushil Satish Chhapekar, Jharna Das, Ajay Kumar, Satish Kumar Yadava, Mukesh Nitin, Vijaya Brahma, Suresh K. Abraham, Nirala Ramchiary

**Affiliations:** 1 Translational and Evolutionary Genomics Lab, School of Life Sciences, Jawaharlal Nehru University, New Delhi, India; 2 Department of Biological Science, Gauhati University, Guwahati, Assam, India; 3 Department of Plant Science, School of Biological Sciences, Central University of Kerala, Periya, Kasaragod, Kerala, India; 4 Centre for Genetic Manipulation of Crop Plants, University of Delhi South Campus, Benito Juarez Road, New Delhi, India; 5 School of Computational and Integrative Sciences, Jawaharlal Nehru University, New Delhi, India; 6 School of Life Sciences, Jawaharlal Nehru University, New Delhi, India; Huazhong University of Science and Technology, CHINA

## Abstract

Bhut jolokia, commonly known as Ghost chili, a native *Capsicum* species found in North East India was recorded as the naturally occurring hottest chili in the world by the Guinness Book of World Records in 2006. Although few studies have reported variation in pungency content of this particular species, no study till date has reported detailed expression analysis of candidate genes involved in capsaicinoids (pungency) biosynthesis pathway and other fruit metabolites. Therefore, the present study was designed to evaluate the diversity of fruit morphology, fruiting habit, capsaicinoids and other metabolite contents in 136 different genotypes mainly collected from North East India. Significant intra and inter-specific variations for fruit morphological traits, fruiting habits and 65 fruit metabolites were observed in the collected *Capsicum* germplasm belonging to three *Capsicum* species i.e., *Capsicum chinense* (Bhut jolokia, 63 accessions), *C*. *frutescens* (17 accessions) and *C*. *annuum* (56 accessions). The pungency level, measured in Scoville Heat Unit (SHU) and antioxidant activity measured by 2, 2-diphenyl-1-picrylhydrazyl (DPPH) free radical scavenging assay showed maximum levels in *C*. *chinense* accessions followed by *C*. *frutescens* accessions, while *C*. *annuum* accessions showed the lowest value for both the traits. The number of different fruit metabolites detected did not vary significantly among the different species but the metabolite such as benzoic acid hydroxyl esters identified in large percentage in majority of *C*. *annuum* genotypes was totally absent in the *C*. *chinense* genotypes and sparingly present in few genotypes of *C*. *frutescens*. Significant correlations were observed between fruit metabolites capsaicin, dihydrocapsaicin, hexadecanoic acid, cyclopentane, α-tocopherol and antioxidant activity. Furthermore, comparative expression analysis (through qRT-PCR) of candidate genes involved in capsaicinoid biosynthesis pathway revealed many fold higher expression of majority of the genes in *C*. *chinense* compared to *C*. *frutescens* and *C*. *annuum* suggesting that the possible reason for extremely high pungency might be due to the higher level of candidate gene(s) expression although nucleotide variation in pungency related genes may also be involved in imparting variations in level of pungency.

## Introduction

The Chili peppers belonging to the family Solanaceae and genus *Capsicum* shows an incredible diversity and are consumed by a large section of population throughout the world because of its impressive health beneficial chemical compounds such as capsaicinoids, carotenoids (provitamin A), flavonoids, vitamins (Vitamins C and E), minerals, essential oils and aroma of the fruits [1,2,3,4,5]. These compounds have shown to possess anticancer [6,7,8,9] anti-inflammatory [10], antimicrobial [11] and antioxidant [12] properties.

Capsaicinoid contents, a group of alkaloids, specifically present only in the members of the genus *Capsicum*, is responsible for giving pungency or heat to the fruit. The capsaicinoid biosynthesis involves convergence of two pathways i.e. the phenylpropanoid pathway which provides the precursor phenylalanine for the formation of vanillylamine, and the branched chain fatty acid pathway which provides the precursors valine or leucine for 8-methyl-6-nonenoyl-CoA formation. Capsaicinoids accumulation occurs specifically in the epidermal layer, called dissepiment of the placental tissue, mostly after 20 to 30 days of pollination and continues till the fruit ripening stage [13,14]. Till date, 23 capsaicinoid analogues have been reported, among which, capsaicin (trans-8-methyl-N-vanillyl-6-nonenamide) and dihydrocapsaicin (8-methyl-N-vanillylnonanamide) constitute about 77–98% of the capsaicinoids content in capsicum [15,16,17]. Apart from these two major capsaicinoids, other capsaicinoids such as nordihydrocapsaicin, homocapsaicin, homodihydrocapsaicin, and nonivamide are also found in small quantities in capsicum fruits [18,19].

Some of the genes involved in capsaicinoid biosynthesis have been characterized and their sequence analysis and expression profiles are studied extensively in different pungent and non-pungent varieties, mostly in *C*. *annuum* [14,20,21,22]. Stewart et al. (2005) [14] reported that the presence of capsaicinoids is controlled by the *Pun1* locus, and confirmed their presence in the interlocular septa of pungent fruit by using HPLC analysis. Later, Stewart et al. (2007) [23] identified a 2.5 kb deletion in *C*. *annuum* sequence that constituted 1.8 kb of the promoter and 0.7 kb of the first exon of SB2-66 clone and named as *Pun1* or *AT3* as it contains acyltransferase domains. Recently, two separate groups i.e. Kim et al. (2014) [24] and Qin et al. (2014) [25] independently published the whole genome sequence and reported capsaicinoid biosynthesis genes in *C*. *annuum*. Reddy et al. (2014) [26] based on candidate gene association mapping studies suggested that the *Pun1* acts as a key regulator in the capsaicinoid pathway and only the expression of this gene decides the accumulation of capsaicinoids. Their analysis also revealed that the *CCR* (Cinnamoyl CoA reductase) and *KAS*I (β-ketoacyl carrier protein synthase I) are the two important enzymes involved in pathways for the regulation of capsaicinoid biosynthesis in capsicum.

Of the total 38 *Capsicum* species reported, *C*. *annuum* is the most extensively grown worldwide among the 6 cultivated species. The other cultivated species are *C*. *baccatum*, *C*. *chinense*, *C*. *frutescens*, *C*. *pubescens*, and *C*. *assamicum* [27,28]. It is believed that the unique climatic condition of North East India have made this region one of the biodiversity hotspots of the world and Bhut jolokia or “Ghost chili” (Assamese word) with its fiery hot pungent characteristics is one of them. It is also known as the Naga King chili or Naga morich in Nagaland and “Umorok” in Manipur State of North east India and is considered as the world’s naturally originated hottest chili (Guinness Book of World records, 2006) [29]. This particular species of pepper which is grouped into *C*. *chinense* is grown mostly in the backyard of North East India household since time immemorial, although recently it is being cultivated commercially because of its unique aroma, nutritive and medicinal properties. Apart from this species, wide variation observed in capsicum germplasm belonging to *C*. *annuum* and *C*. *frutescens* makes North Eastern India one of the important sources of genetic resources of chili peppers. However, only fragmented studies and reports on diversity in capsicum germplasm, which is based on capsaicinoids are currently available [30,31]. Recently, Islam et al. (2015) [32] evaluated the levels of variation in capsaicinoid content in 139 diverse accessions using high performance liquid chromatography (HPLC) method. However, the detailed characterization and documentation of capsicum germplasm with respect to morphological traits, pungency, other metabolites, vitamins and their contribution towards antioxidant activities have not been reported till date. Furthermore, extensive comparative studies on expression of candidate genes involved in capsaicinoid biosynthesis using germplasm belonging to different capsicum species of North Eastern India are lacking.

Therefore, in the present study, our main objectives were to i) characterize different genotypes of the three species—*C*. *chinense*, *C*. *frutescens* and *C*. *annuum* for fruit morphology and metabolites including pungency, vitamins, and antioxidant activity; ii) to understand the overall correlation between different metabolites and antioxidant activities; and iii) to compare the pungency related candidate gene expression in contrasting capsicum germplasm belonging to different capsicum species and their correlations with pungent phenotypes.

## Materials and Methods

### Plants materials

Majority of the 136 genotypes belonging to the three capsicum species (*C*. *chinense*, *C*. *frutescens*, and *C*. *annuum*) were collected from different regions of North East India i.e. Assam, Nagaland, Manipur and Meghalaya and grown in an experimental plot of School of Life Sciences, Jawaharlal Nehru University, New Delhi following standard cultivation practices. Few samples of *C*. *annuum* were collected from the states of Kerala, Jammu and Kashmir, and Delhi. Since the collections of germplasm were done from traditional market places, no permission was required. Furthermore, no restricted or endangered materials were damaged during sample collection and research activities. The geographical coordinates are provided in S1 Table. These 136 genotypes included 63 (Acc 1–63) genotypes from Bhut jolokia (*C*. *chinense*), 17 (Acc 64–80) genotypes from *C*. *frutescens* and 56 (Acc 81–136) genotypes from *C*. *annuum*. The Capsicum plants were grown during May to December, 2014 in sunny days in experimental research field with well drained loamy soils rich in nutrients. The seeds were treated with Bavistin and Sodium hypochlorite to prevent seed-borne diseases and sown in germination tray. The field is prepared with repeated plowing. Before sowing the field was sprayed with copper fungicide to prevent damping off and to control thrips. A 35 kg P (phosphorus) per hectare and 35 kg K (potash) per hectare was applied. The healthy seedlings of 1 months old were transplanted with spacing of 45 cm X 50 cM (plant to plant and row to row). A 70 kg of N (nitrogen) per hectare was applied at 30, 60, 90 days after transplanting for flowering and proper vegetative growth. The field is irrigated once in 4–5 days. The plants were grown in three rows, each of 3 meter length and 6–10 fruits (depending on the size) from middle plants and second flush of fruit settings were harvested carefully at ripening (mature) stage and kept for drying for further analysis.

### Reagents & chemicals

The entire chemicals used in this study were HPLC grade and purchased from Himedia (India) and Sigma Aldrich Co. (USA). The standards of capsaicin and dihydrocapsaicin for estimation of capsaicinoid content and 2, 2-diphenyl-1-picrylhydrazyl (DPPH) for antioxidant assays were purchased from Sigma Aldrich Co. (USA).

### Capsaicinoid and other metabolite extractions

The ripened fruits (deseeded) were homogenized in methanol (1:10, w/v) and filtered through Whatman paper No. 1 over anhydrous Sodium sulphate (Na_2_SO_4_). The filtered extract was evaporated to dryness in vacuum and the residue was suspended with 10 ml acetonitrile as reported earlier [33]. The samples were then centrifuged at 14,000 rpm for 10 minutes and filtered through 0.45 μm Polytetrafluoroethylene (PTFE) membrane filter (Millipore) before injecting to GC-MS. Three independent replicates of samples were used for extraction and GC-MS analysis.

### GC-MS analysis

Detection and quantification of capsaicinoids and the presence of other metabolites was carried out by gas chromatography coupled with mass spectrometry (Shimadzu QP2010 Plus) equipped with a Rtx- 5 MS capillary column (0.25 mm film thickness, 0.25 mm internal diameter, and 30 m in length). The oven temperature was set at 100°C for 2 min, then increased to 250°C at a rate of 5°C per minute, and finally to 280°C at a rate of 10°C per minute. One μl of each sample was injected to the column in split mode (split ratio 10) with helium as the carrier gas with a flow rate of 1.21 ml per minute. The presence of distinctive peak fragmentation patterns for various metabolites was detected by an MS detector in full scan mode. Capsaicin and dihydrocapsaicin were determined using external reference standards injected under the same conditions. Their identification was based on the retention times and mass measured under identical GC-MS conditions, while their quantitative determinations in the different samples were carried out using the peak areas. Identification of metabolites was confirmed by comparing the spectral data of peaks with the corresponding standard mass spectra from the library database [National Institute of Standards and Technology library (NIST05) and Wiley 8]. Capsaicinoid contents from all the genotypes were expressed in μg/g of fruits and final value was expressed as Scoville heat unit (SHU) by multiplying with the conversion factor of 16.0 x 10^6^ for capsaicin and dihydrocapsaicin, 9.3 x 10^6^ for nordihydrocapsaicin and 9.2 x 10^6^ for nonivamide [34].

### Antioxidant assay

Antioxidant activity of different capsicum species was evaluated by 2, 2 diphenyl-1-picrylhydrazyl (DPPH) free radical scavenging assay. The DPPH solution (100 μM) was freshly prepared in 100% methanol. Sample solutions (concentrations: 100mg/ml, 50mg/ml and 25mg/ml) were prepared in acetonitrile and 25 μl aliquots were then added to a 96 well micro plate containing a 225 μl DPPH (0.1 mM). The reaction mixtures were incubated in the dark, at room temperature for 15 minutes and the absorbance was measured at 517 nm in a Multi-plate reader (Thermo Fisher Scientific). The free radical scavenging capability was evaluated by comparing to a blank, which contained only methanol. For obtaining the calibration curve, five concentrations of ascorbic acid (100μg– 6.25μg) and capsaicin (1000μg– 62.5μg) in acetonitrile were used. Percentage of free radical scavenging activity (AA) was determined by using the following equation-

$$\%\text{AA}=\frac{\left(\text{Acontrol}-\text{Asample}\right)}{\text{Asample}}\times 100$$

Where A_Control_ is the absorbance of the reaction mixture excluding test sample (DPPH solution) and A_Sample_ is the absorbance of the reaction mixture of the test sample (DPPH solution with sample). All the tests were conducted in triplicates and the values were expressed as means ± SD [35].

### Quantitative Real-Time PCR

Total RNA was extracted using Lithium chloride (LiCl) precipitation method from fruit tissue at green, breaker and mature stages of fruit development. Complementary DNA (cDNA) was synthesized from 1 μg of RNA using Verso cDNA synthesis kit (Thermo Fisher Scientific) according to manufacturer’s instructions. To perform expression analysis, genes from the capsaicinoid pathway were selected and primers were designed using Primer Express version 3.0 software (Applied Biosystems) and custom synthesized from Sigma Genosys (Sigma Aldrich). Using these primer pairs, qRT-PCR was performed in 10μl reaction volumes that contained 1μl cDNA, 5 μl SYBR green master mix (Agilent Technologies), 0.2 μl of 10 μM of each primer, 0.2 μl of the reference dye (Agilent Technologies) and 3.4 μl of nuclease free water. qRT-PCR was performed in a ABI7500 Fast Real-Time PCR system (Applied Biosystems) with the following thermal profile: initial denaturation at 95°C for 2 min followed by 40 cycles of amplification of 15 sec at 95°C and 1 min at 60°C. Finally, a melting curve analysis was performed from 60 to 95°C in increments of 0.5°C to confirm the presence of a single product and absence of primer dimers. Each sample was assayed in triplicates, and each experiment was repeated at least twice. For expression analysis, comparative threshold cycle (Ct) method was used which also called as 2^−[ΔΔCt]^ method [36]. For data normalization a house keeping gene actin was used as an internal control.

### Statistical analysis

Summary statistics and principal component analysis (PCA) of the metabolites obtained from GC-MS analysis of the *Capsicum* genotypes were performed using the mixOmics package in R environment for statistical computing (version 3.2.3). Summary statistics comprised of mean, standard deviation and analysis of variance (ANOVA) at 95% confidence limit, F-value (P≤ 0.001) significance. Correlation analysis using Pearson correlation method and adjusted for multiple testing by using Bonferroni correction were implemented in R (S2 Table). Student’s t-test was used for analyzing qRT-PCR data.

## Results

### Morphological variations

**Fig 1:**
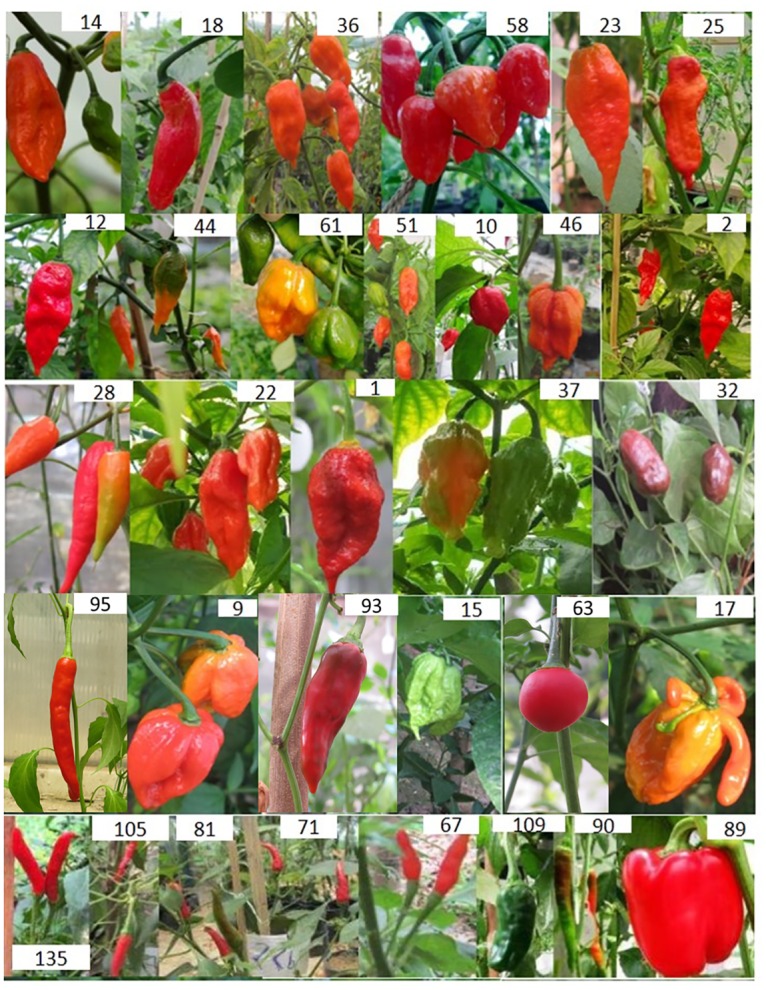
Morphological diversity of *Capsicum* species. Selected *Capsicum* germplasm from North East India showing contrasting phenotypes for fruit morphology, color, and fruiting habits. Accessions in 1-3^rd^ rows are contrasting Bhut jolokia genotypes (*C*. *chinense*), 4^th^ and 5^th^ row contains *C*. *chinense*, *C*. *frutescens* and *C*. *annuum* accessions.

The 136 different accessions collected mainly from North Eastern India were characterized for fruiting habits, fruit morphology and colors (Fig 1 and Table 1). The highest variations of fruit morphology, especially fruit shape, size and length were observed in *C*. *annuum* accessions followed by *C*. *chinense*, while *C*. *frutescens* showed mostly one type of fruit shape among the collected accessions. The fruit shapes observed were long, elongate, ovate, round, pumpkin shape and varied from small to large fruits in *C*. *annuum*; ovate and elongated type in *C*. *chinense*; and very small elongated fruits in *C*. *frutescens*. The contrast in fruit color varied from orange, red, yellow and chocolate colors. Fruiting habits were observed to be upright and pendant in *C*. *annuum*, only upright in *C*. *frutescens* and only pendant in *C*. *chinense* (Bhut jolokia). Variation from single to bunch type fruiting habits were observed in *C*. *annuum* and *C*. *chinense*, whereas in *C*. *frutescens* only single fruiting habit was observed.

**Table 1:** Morphological characteristics of *Capsicum* fruits.

Species	Fruit length (cm)			Fruit weight (g)			Seed count number			Seed weight (g)	Fruit characteristics			
	Mini-mum	Maxi-mum	Ave-rage	Mini-mum	Maxi-mum	Ave-rage	Mini-mum	Maxi-mum	Ave-rage	Ave-rage	Fruiting habit	Fruit shape	Fruit color at maturity	Fruit shape at blossom end
*C*. *annuum*	1.3	10.22	5.59	0.22	7	2.89	14	100	46.75	0.071	Mostly pendant	Elongated, almost round or block shaped	Light red, yellow, dark red	Pointed, blunt or sunken
*C*. *chinense*	2.7	8	4.73	0.7	10.58	4.98	8	60	26.11	0.035	Mostly pendant	Triangular, ovate	Red, Orange or chocolate	Pointed, blunt or sunken
*C*. *frutescens*	0.7	2.56	1.4	0.05	0.42	0.28	3	14	7.07	0.053	Erect upward	Short slender	Red or Orange	Pointed or blunt

### Determination of capsaicinoid contents

Pungency, a unique and important property of *Capsicum* species, attributed to the capsaicinoid contents was analyzed in all the 136 different germplasm collected using Gas Chromatography coupled with Mass Spectrometry (GC-MS). The extraction was carried out by using acetonitrile and the capsaicinoid contents was separated by using GC-MS. The quantity of the complex was calculated by means of calibration curves. The correlation coefficients for capsaicin and dihydrocapsaicin were >0.998 and >0.995, respectively (S1 Fig). Of the two major capsaicinoids, i.e. capsaicin and dihydrocapsaicin, the former was found to be more abundant in the collected *Capsicum* germplasm belonging to *C*. *annuum*, *C*. *frutescens* and *C*. *chinense* of North East India. Other two capsaicinoids, nordihydrocapsaicin and nonivamide were also present in many of the accessions but in small quantities. Capsaicinoid contents were measured both in Scoville Heat Unit and amount in μg/g of fruit for all the genotypes (S3 Table).

**Fig 2:**
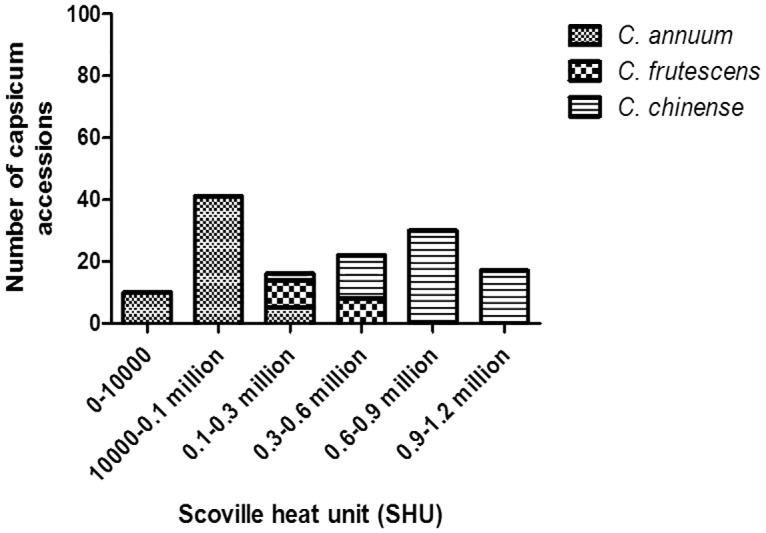
SHU range of different *Capsicum* species. Total capsaicinoids content observed in *C*. *chinense* (63 accessions), *C*. *frutescens* (17 accessions) and *C*. *annuum* (56 accessions) accessions in Scoville Heat Unit (SHU).

The pungency, as expected was observed to be high in *C*. *chinense* accessions compared to accessions belonging to the other two *Capsicum* species. The Scoville Heat Unit (SHU) value, a unit of heat/pungency measurement, ranged from 272897 (0.27 million) to 1037305 (1.0 million), 109508 (0.1 million) to 487619 (0.48 million) and 0 (bell pepper) to 203731 (0.2 million) in *C*. *chinense*, *C*. *frutescens* and *C*. *annuum* accessions, respectively (S3 Table and Fig 2). The highest pungency of more than 1 million SHU value was obtained for three *C*. *chinense* genotypes with accession numbers 8, 23 and 42. SHU values between 0.9 to 1.0 million were observed in 14 genotypes of *C*. *chinense* (Accessions 7, 11, 19, 20, 22, 24, 25, 29, 32, 43, 45, 48, 50 and 54), between 0.8 to 0.9 million in 10 genotypes (Acc 2, 6, 10, 18, 31, 40, 41, 49, 53 and 56), 0.7 to 0.8 million in 8 genotypes (Acc 4, 12, 16, 17, 34, 37, 53 and 55), 0.6 to 0.7 million in 10 genotypes (Acc 1, 14, 15, 28, 30, 38, 44, 46, 47 and 51), 0.5–0.6 million in 10 genotypes (Acc 5, 9, 13, 21, 26, 33, 35, 36, 59 and 63) and only 8 genotypes of *C*. *chinense* (Acc 3, 27, 39, 57, 58, 60, 61 and 62) showed pungency below 0.5 million with varying capsaicin and dihydrocapsaicin levels. Most of the genotypes from *C*. *frutescens* showed moderate pungency with a SHU value ranging between 0.3–0.5—million, but 9 genotypes expressed a pungency level below 0.3 million. The *C*. *annuum* accessions 95, 98, 116, and 126 exhibited the lowest pungency level (<5000 SHU). Nordihydrocapsaicin and nonivamide peaks were absent in many of the analyzed genotypes of *Capsicum*. As expected, the accession 87 (bell pepper) showed zero pungency.

### Analysis of other metabolites

**Fig 3:**
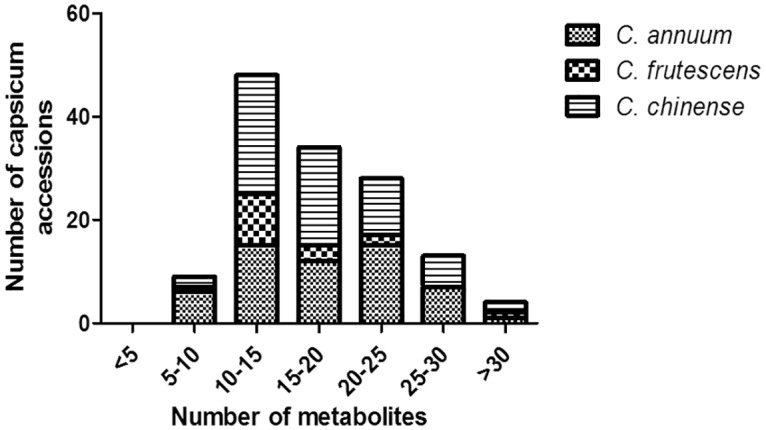
Metabolite range of different *Capsicum* species. Metabolite range of *C*. *chinense*, *C*. *frutescens* and *C*. *annuum* varieties.

In the current study, apart from the capsaicinoid contents, other metabolites were also analyzed using GC-MS. These 61 different metabolites were identified after acetonitrile extraction of dried capsicum fruit. These metabolites comprises carboxylic acids (such as Propanoic acid, butanoic acid, hexanoic acid etc.), fatty acid and esters (such as Decanoic acid, Palmitic acid etc.), hydrocarbons (Cyclopentane, Naphthalene etc.), aldehydes (Tetradecanoic acid, Pentadecanoic acid, Eicosanoic acid), terpenoids (2,7-Octadiene, Geranyl linalool isomer B), Alcohol (hexanol, isopropanol) and Vitamin E (α-tocopherol). However, the metabolites concentration varied with genotypes (S4 Table). Many of the compounds were found only in specific genotypes. *C*. *chinense* and *C*. *annuum* exhibited a slightly higher number of metabolites compared to *C*. *frutescens*. An average of 17, 14 and 17 metabolites was identified in the genotypes belonging to *C*. *chinense*, *C*. *frutescens* and *C*. *annuum*, respectively (Fig 3). The number of metabolites identified ranged from 7–32 for *C*. *chinense*, 5–31 for *C*. *annuum* and 9–32 for *C*. *frutescens*, respectively. The metabolites like benzoic acid hydroxyl esters, which are identified in large percentage in majority of *C*. *annuum* genotypes, were totally absent in the *C*. *chinense* genotypes and present sparingly in few genotypes of *C*. *frutescens*. Other metabolites like fatty acids and corresponding esters, hydrocarbons, aldehydes, alcohols and terpenoids were randomly distributed in all the genotypes of *C*. *chinense*, *C*. *frutescens* and *C*. *annuum*.

### Antioxidant activity of different *Capsicum* genotypes

**Fig 4:**
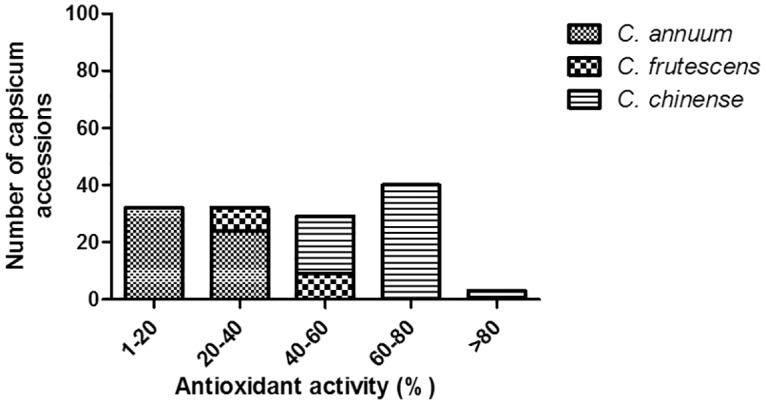
Range of antioxidant activity of different *Capsicum* species. Anti-oxidant activity using DPPH assay obtained for *C*. *chinense*, *C*. *frutescens* and *C*. *annuum* varieties and represented in 25mg/ml dilutions.

The antioxidant activity of different *Capsicum* varieties were analyzed by determining the DPPH scavenging capability. Significant differences in antioxidant activity were observed between *C*. *chinense*, *C*. *frutescens* and *C*. *annuum* accessions. The highest antioxidant (free radical scavenging) activity was observed in *C*. *chinense* accessions compared to *C*. *frutescens* and *C*. *annuum* accessions. The antioxidant activity determined by DPPH assay ranged from 40% to 83% in *C*. *chinense* accessions followed by 31% to 50% in *C*. *frutescens* and 3% to 38% in *C*. *annuum* accessions. The average free radical scavenging activity from all the genotypes of *C*. *chinense*, *C*. *frutescens* and *C*. *annuum* were 63.83 ± 2.21, 40.76 ± 3.72 and 18.63 ± 4.52 respectively. Three accessions of *C*. *chinense* showed more than 80% antioxidant activity, while 39 accessions exhibited antioxidant activity between 60 to 80%, and 21 accessions had antioxidant activity between 40 to 60%. The genotypes from *C*. *frutescens* showed antioxidant activity ranging from 20 to 50%, 8 accessions showed 20 to 40%, while 9 accessions showed 40–50% antioxidant activities. The majority of *C*. *annuum* genotypes exhibited antioxidant activity less than 20% (Fig 4 and S3 Table).

### Principal component analysis

**Fig 5:**
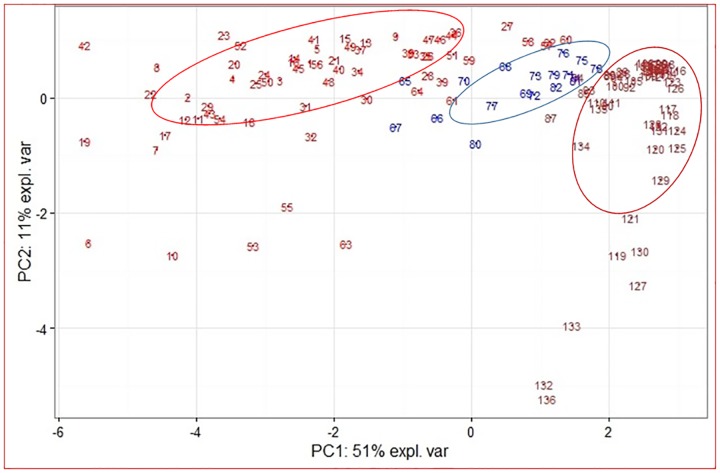
Principal component analysis of metabolites identified using gas chromatography–mass spectrometry (GC–MS) analysis. For GC–MS, different genotypes of *C*. *chinense* [Acc 1–63 (major accession formed red circle)], *C*. *frutescens* [Acc 64–80 (major accession formed blue circle)] and *C*. *annuum* [Acc 81–136 (major accession formed brown circle)] were analysed and the correlation variances explained by the PC1 and PC2 components are 51% and 11%, respectively.

Metabolite profiling of capsicum fruits identified a total of 65 metabolites by GC-MS. Of these, the metabolites which were present in almost all 136 genotypes were selected for further analysis. These include various bioactive fatty acids like palmitic acid (hexadecanoic acid), octadeccanoic acid (stearic acid), 9(Z)-octadecenoic (oleic acid), cyclopentane and n-octacetylamide and alkaloids like capsaicin, dihydrocapsaicin, nordihydrocapsaicin and nonivamide and α-tocopherol (vitamin E). These metabolites play different roles in capsaicinoid biosynthesis pathway, maintaining cell membrane integrity, signaling and defense mechanism etc. The Principal component analysis (PCA) revealed that genotypes could be differentiated based on their metabolite profiles and the correlation variances explained by the two principal components (PC 1 and PC 2) were observed to be 51% and 11%, respectively (Fig 5). Even though majority of accessions from *C*. *chinense* and *C*. *annuum* fall in to separate clusters, the patterns of metabolite expression across the 136 genotypes were not completely differentiated based on the type of species (*C*. *chinense*, *C*. *frutescens* and *C*. *annuum*).

Correlation analysis of these 10 metabolites along with the antioxidant activity showed high correlation among the metabolites with their antioxidant activity. The correlation analysis showed significant correlations between many of these metabolites (Table 2 and S2 Table). Correlation circle plot of PCA analysis clearly illustrated that, there exists correspondence between capsaicin, dihydrocapsaicin, hexadecanoic acid, cyclopentane, α-tocopherol and antioxidant activity (S2A Fig). Metabolites forming a cluster were projected in the same direction with significant distances from the origin highlighting the strength of correlation. In addition PCA also revealed a similar pattern of metabolite correlation across the *Capsicum* genotypes from *C*. *chinense*, *C*. *frutescens* and *C*. *annuum* (S2B Fig).

**Table 2:** Pearson correlation co-efficient values observed between different metabolites on the basis of their peak intensities.

	Stearic acid	Oleic acid	Cyclopentane	n-octacetylamide	Capsaicin	Dihydrocapsaicin	Nordihydrocapsaicin	Nonivamide	Vitamin E	Antioxidant activity
**Palmitic acid**	-0.045	0.64***	0.41***	0.449***	0.782***	0.746***	0.327**	0.341**	0.715***	0.800***
**Stearic acid**		-0.044	0.07	0.065	-0.069	-0.08	-0.068	0.122	0.131	-0.054
**Oleic acid**			0.311*	0.561***	0.703***	0.601***	0.421***	0.087	0.426***	0.699***
**Cyclo pentane**				0.601***	0.351**	0.354**	0.066	0.093	0.307*	0.412***
**n-octacetylamide**					0.604***	0.449***	0.101	0.162	0.298	0.421***
**Capsaicin**						0.741***	0.261	0.212	0.647***	0.913***
**Dihydrocapsaicin**							0.442***	0.264	0.577***	0.832***
**Nordihydrocapsaicin**								0.224	0.293*	0.345**
**Nonivamide**									0.268	0.302*
**Vitamin E**										0.683***

* represents significant at 0.05 level

** represents significant at 0.01 level

*** represents significant at 0.001 level

### Expression analysis of candidate genes

**Fig 6:**
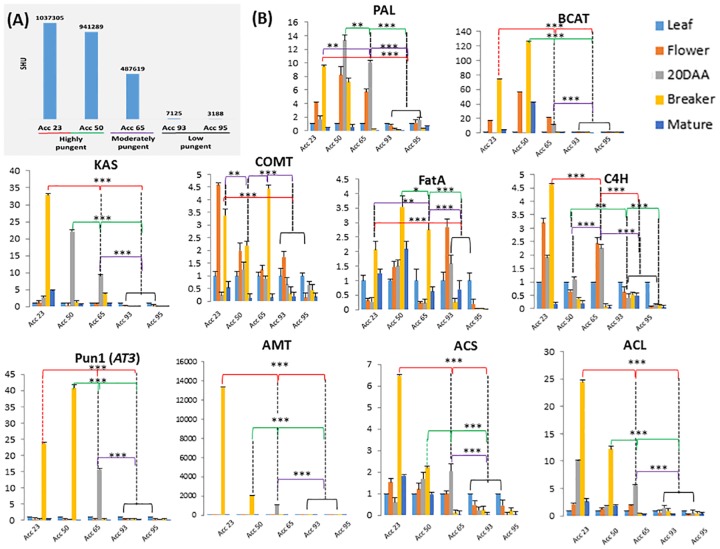
Pungency and capsaicinoid biosynthesis gene expression analysis. **(A)** Pungency analysis of selected *Capsicum* genotypes **(B)** Quantitative real time PCR analysis to analyze the expression of candidate genes involved in capsaicinoid biosynthesis pathway in highly pungent Bhut jolokia (Acc 23 and Acc 50), moderately pungent *C*. *frutescens* (Acc 65) and low pungent *C*. *annuum* (Acc 93 and Acc 95) accessions. The expression analysis was done in leaf, flowers, and three different stages of fruit developmental i.e. green (20 days after anthesis), Breaker (30–45 days after anthesis) and Mature stage of each genotype. The majority of the important genes involved in the capsaicinoid biosynthesis pathway (*Pun 1*, *AMT*, *ACS*, *ACL*, *KAS* and *BCAT*) were expressed very high in *C*. *chinense* accessions followed by *C*. *frutescens*. The low pungent *C*. *annuum* accessions showed very low expression of these genes. The other genes (*PAL*, *COMT*, *FatA* and *C4H*) were expressed variably among the three species. ***P<0.001

The different *Capsicum* accessions collected from North East India showed wide variation in pungency contents as evidenced from biochemical analysis, and since the genes involved in capsaicinoid biosynthesis pathway (S3 Fig) have been reported in *C*. *annuum*, we made attempts to identify variations in the expression of candidate genes in accessions with contrasting pungency levels. In the present study, 10 candidate genes of the capsaicinoid biosynthesis pathway were selected to analyze their expression patterns in leaf, flower and three stages of fruit development (green, breaker and mature stages of the fruit) in highly pungent (*C*. *chinense* accessions 23 and 50), moderately pungent (*C*. *frutescens* accession 65) and low pungent genotypes (*C*. *annuum* accessions 93 and 95) (Fig 6A). The candidate genes selected were *PAL* (phenylalanine ammonia-lyase), *C4H* (cinnamate 4-hydroxylase), *COMT* (caffeic acid 3-O-methyltransferase), *ACL* (acyl-CoA synthetase), *pAMT* (putative aminotransferase), *BCAT* (branched-chain amino acid aminotransferase), *KAS* (ketoacyl-ACP synthase), *ACL* (malonyl-acyl carrier protein), *FAT* (acyl-ACP thioesterase) and *AT3* (*Pun1* or acyltransferase). The sequences of forward and reverse primer pairs are listed in (S5 Table). We observed that the expression levels of these genes varied with the genotypes having different pungency levels. The majority of genes showed significantly higher expression in *C*. *chinense* genotypes followed by moderately high pungent *C*. *frutescens*, whereas a very low level of expression was observed in *C*. *annuum* genotypes (low-pungent). Amongst these candidate genes, *pAMT*, *Pun1/AT3*, *PAL* from Phenylpropanoid pathway and *BCAT*, *KAS* and *ACL* from Fatty acid biosynthetic pathway were found to be highly expressed in pungent genotypes especially in breaker stage of the fruit development (Fig 6B).

## Discussion

The North Eastern region of India, one of the biodiversity hotspot of the world, harbors many of the endangered and endemic species of plants and animals. The unique climatic conditions of this region have also favored the evolution of *Capsicum* species, thereby producing landraces and traditional cultivars with diverse morphology, fruiting habits, metabolite contents with varied levels of biotic and abiotic resistances. Bhut jolokia or Ghost chili, reported by the Guinness Book of World Records as the naturally occurring world`s most pungent chili pepper has also evolved in this region. Although this particular *Capsicum* species have been cultivated from time immemorial in the kitchen gardens of North East India, until recently, no systematic commercial cultivation was practiced. Having enormous commercial potential of this crop particularly in producing spices, natural color and for its potential use in medicine, the systematic characterization and identification of potential germplasm for future use in breeding program is pre-requisite. The fragmented studies that have been reported so far are based on the analysis of pungency contents of only few genotypes. Importantly, detailed analysis of fruit metabolites and antioxidant activity and their correlations have not been reported. Furthermore, experimental designs understanding the fiery hot property of this species have also not been reported. We collected 136 different capsicum landraces and traditional cultivars mainly from different places of North East India where chili peppers are grown. As expected we observed a variation in fruit morphology reflected in fruit size, shape, length and color. The different fruit colors (orange, yellow, red etc.) are reported to be determined by both the amount and composition of carotenoids and pigments. The yellow-orange color of peppers is formed by *α*- and *β*-carotene, zeaxanthin, lutein, and *β*-cryptoxanthin and the red color of peppers is due to the presence of carotenoid pigments of capsanthin, capsorubin, and capsanthin 5,6-epoxide [37]. Of the many genes reported, only phytoene synthase (*Psy*) and capsanthin–capsorubin synthase (*Ccs*) are directly shown to be correlating with red, yellow and orange color in different allelic combinations, and studies to identify more genes imparting color are underway. However, no concrete evidence of gene(s) imparting chocolate color is reported in *Capsicum* species. The identification of chocolate color Bhut jolokia in the present study provides new opportunity to use this genotype in future study in identifying genes imparting chocolate color.

GC-MS data demonstrated the different capsaicinoid levels in 136 C*apsicum* genotypes. Our analysis showed that capsaicin and dihydrocapsaicin represented the major fractions compared to other capsaicinoid components, which is consistent with the results obtained in several published reports [38,39,40]. In addition, the present study confirms that capsaicin is the primary capsaicinoid component in almost all of the analyzed *Capsicum* genotypes. However, the total capsaicinoids concentration showed intra-specific as well as inter-specific variations as reported earlier [41,42,43,44,45,46]. The present study identified 17 genotypes from *C*. *chinense* group with more than 0.9 million SHU which is distinctly higher than the previously reported pungency of Habanero [47,48,49]. Although, Bhut jolokia (*C*. *chinense*) is known as the natural hottest chili pepper, low pungency Bhut jolokia genotypes were also observed (with pungency as low as 272897.1 ± 38759 SHU), suggesting that during the course of evolution, low pungency alleles were formed and accumulated in those genotypes. Another plausible reason for the low pungency in these accessions could be attributed to the crossability of Bhut jolokia with other cultivated species and therefore, cross pollinations in nature with low pungent *C*. *annuum* followed by selection might have developed low pungent Bhut jolokia genotypes. A recent study by Dubey et al. (2015) [30] also reports quantification of capsaicin content of 25 *Capsicum* genotypes from North Eastern states of India by using spectrophotometry in which they have also reported variations in pungency content. However, in our study we have listed and quantified all the components of capsaicinoids (capsaicin, dihydrocapsaicin, nor dihydrocapsaicin and nonivamide). The present study also shows the high antioxidant activity of Bhut jolokia accessions compared to the other two species suggesting a strong correlation between capsaicinoids and antioxidant activity. This was further supported by the fact that *Capsicum* accessions possessing lower capsaicin and dihydrocapsaicin also exhibited the lowest antioxidant activity thereby indicating that capsaicinoids also contributes in reduction of free radicals, a property which is deemed beneficial for human health. This finding is also supported by recently published data of Sora et al. (2015) [50].

GC-MS is one of the established and highly suitable techniques for metabolite profiling, as it combines a highly efficient separation technique with versatile and sensitive mass detection methods [51,52]. We have used GC-MS to determine the level and composition of fruit metabolites in Bhut jolokia, in which except for the capsaicinoid contents other health beneficial fruit metabolites are largely uncharacterized. We identified a total of 65 metabolites including the four capsaicinoids components, fatty acids and esters, aliphatic esters and aldehydes, alcohols, hydrocarbons and vitamin E. These metabolites have been previously reported to be found in various ripened *Capsicum* varieties mostly in *C*. *annuum* [53,54], and are the primary components of the essential oils, which give aroma to chili peppers [1,4], but have not been characterized in Bhut jolokia. These metabolites were found in varied concentrations in the different genotypes and species. These health promoting compounds of Bhut jolokia can be studied in detail and manipulated in future breeding programs.

Among the total of 65 metabolites, 10 were detected in majority of the *Capsicum* genotypes. Principal component analysis showed five metabolites to be highly correlated among them along and to the antioxidant activity. These metabolites are palmitic acid, cyclopentane, capsaicin, dihydrocapsaicin and α-tocopherol. This appears to be the first study showing the positive correlations in fatty acid, capsaicinoid and vitamin E pathway. The present data is also consistent with the previous study, which demonstrates the role of long chain fatty acids in capsaicin biosynthesis pathway in *C*. *annuum* [55,56]. There are other efforts which demonstrate the correlation of vitamin E and capsaicinoid synthesis [57]. None of the studies so far have been reported underlining the correlation between fatty acids, capsaicinoids and vitamin E along with antioxidant activity in a large number of genotypes which comprise of *C*. *chinense*, *C*. *frutescens* and *C*. *annuum* groups. Although, pungency and other metabolites found in *C*. *annuum* genotypes varied, it was not as wide as in the case of Bhut jolokia (*C*. *chinense*) genotypes. These might be due to the fact that most of the *C*. *annuum* varieties are derived from more related genotypes showing homogeneity.

Our qRT-PCR results showed the relationship between the expression of candidate genes and the level of pungency in the *Capsicum* genotypes analyzed in this study. Further, among the different stages of fruit development used in this study, the maximum expression of these genes was obtained at 20 DAA (green in *C*. *frutescens*) and breaker stage (35–45 DAA in *C*. *chinense*). We found many candidate genes i.e. *pAMT*, *Pun1*, *KAS*, *ACS*, *BCAT*, *ACL* and *FAT* to be highly expressed in different fruit developmental stages which were many fold higher in *C*. *chinense* compared to the *C*. *frutescens* and *C*. *annuum* genotypes suggesting a high correlation of gene expression with higher pungency content. Iwai et al. (1979) [13] also observed that capsaicinoids are synthesized in the placenta in between 20 to 30 DAA in pungent varieties of *Capsicum*. Very recently, Ogawa et al. (2015) [58] verified the involvement of *Pun1* genes in capsaicin biosynthesis. They also studied the expression profiles of *Pun1* and *pAMT* genes and concluded that the accumulation of capsaicin content is highly correlated with the expression levels of these genes in different varieties of *Capsicum*. The expression analysis revealed that along with *Pun1*, the *pAMT* gene also significantly expressed very high in 20 DPA and breaker stages of fruit development in *C*. *frutescens* and *C*. *chinense* respectively, compared to low pungent *C*. *annuum*. Lang et al. (2009) [59] observed that functional loss of *pAMT* gene leads to formation of capsinoids (a sweat analog of capsaicinoid) in non-pungent *C*. *annuum* cv. CH-19. A single nucleotide (T) insertion at 1291 bp of *pAMT* resulted in formation of stop codon that prevented gene translation and protein accumulation. The study confirms the crucial role of *pAMT* gene in capsaicinoid biosynthesis pathway. Our result shows that *pAMT* gene is mainly expressed in 20 DPA and breaker stages of fruit and co-related with the amount of pungency. The expression of *pAMT* gene is significantly high in *C*. *chinense* (highly pungent) accessions compared to *C*. *frutescens* (moderately pungent) and *C*. *annuum* (low pungent). An association mapping study of Reddy et al. (2014) [26] revealed that *Pun1* acts as a key regulator of major metabolites and that the capsaicinoids accumulation depends on the expression of *Pun1*. Further, the evidences available support *KAS* as an important player in altering the pungency in *Capsicum* varieties. For e.g. Aluru et al. (2003) [21] reported that *KAS* expression was positively correlated with the level of pungency. Later, this was confirmed by Abraham-Juarez et al. (2008) [60] by a virus induced silencing of *KAS* leading to very low levels of mRNA and thus low capsaicinoids in the pungent variety of *C*. *chinense*. Our study is the first comprehensive study in Bhut jolokia which shows correlation of expression of candidate genes of pungency biosynthesis pathway with the pungency content.

## Conclusions

Diversity is a prerequisite for breeding improved varieties in any crop plant. Our findings from the present study, which observes large morphological and fruit metabolites diversity among the *Capsicum* genotypes found in the North East India, would constitute a valuable resource for future improvements on capsicum breeding. Bhut jolokia, although known as naturally occurring highest pungent chili pepper, also showed to have low pungent genotypes. Our results suggest that the many fold higher expression of candidate genes involved in capsaicinoid biosynthesis pathway is the most plausible reason for finding very high pungent phenotypes of Bhut jolokia compared to *C*. *frutescens* and *C*. *annuum*. Furthermore, the variability found in the nutritionally valuable metabolites including capsaicinoids (pungency), vitamins; and a positive correlation with antioxidant activities suggested that these genotypes would be potential genetic stocks towards improving health promoting *Capsicum* varieties through a combined genetics and genomics approach in future capsicum breeding programs.

## Supporting Information

S1 Fig Calibration curve for capsaicin and dihydrocapsaicin. (TIF)

S2 Fig Correlation circle plot of analysed metabolite in *Capsicum* genotypes. **(A)** Correlation circle plot shows that there is a similar correlation pattern between certain metabolites and antioxidant activity in majority of the *Capsicum* species from (*C*. *annuum*, *C*. *frutescens and C*. *chinense*). These metabolites are identified and represented as M1 (hexadecanoic or palmitic acid), M3 cyclopentane, M6 (capsaicin), M7 dihydrocapsaicin, M10 (α-tocopherol or vitamin E) and AA (antioxidant activity). The strongly correlated metabolites were projected in the same direction from the origin of the circle. The distance from the origin indicates the strong association of the metabolites. (**B)** Biplot analysis showing the association between the *Capsicum* accessions and metabolites. Majority of the accessions from the three species of *Capsicum* exhibited similar pattern of metabolites correlation. The angle between the arrows (vectors) showed inversely proportional to the correlation of metabolites. Highly correlated metabolites point in the same direction; uncorrelated metabolites are at right angles to each other.
(TIF)

S3 Fig Flow chart of capsaicinoid biosynthesis pathway. (TIF)

S1 Table Geographical locations and coordinates of sampling sites. (DOCX)

S2 Table Bonferroni and Benjamini correction table. (XLSX)

S3 Table Concentration of different capsaicinoid components (in μg/g dry weight of fruit) and antioxidant activities observed in *Capsicum* accessions. (DOCX)

S4 Table Average area of different metabolites observed in capsicum accessions belonging to Bhut jolokia (*C*. *chinense*), *C*. *frutescens* and *C*. *annuum*. (XLSX)

S5 Table List of primer sequences used for expression studies of pungency candidate genes. (DOCX)
