# Supplementary figures and images for: Comparative Analysis of Fruit Metabolites and Pungency Candidate Genes Expression between Bhut Jolokia and Other Capsicum Species
Source: PLoS One. 2016 Dec 9;11(12):e0167791. doi: 10.1371/journal.pone.0167791 (PMC5147997; doi:10.1371/journal.pone.0167791)

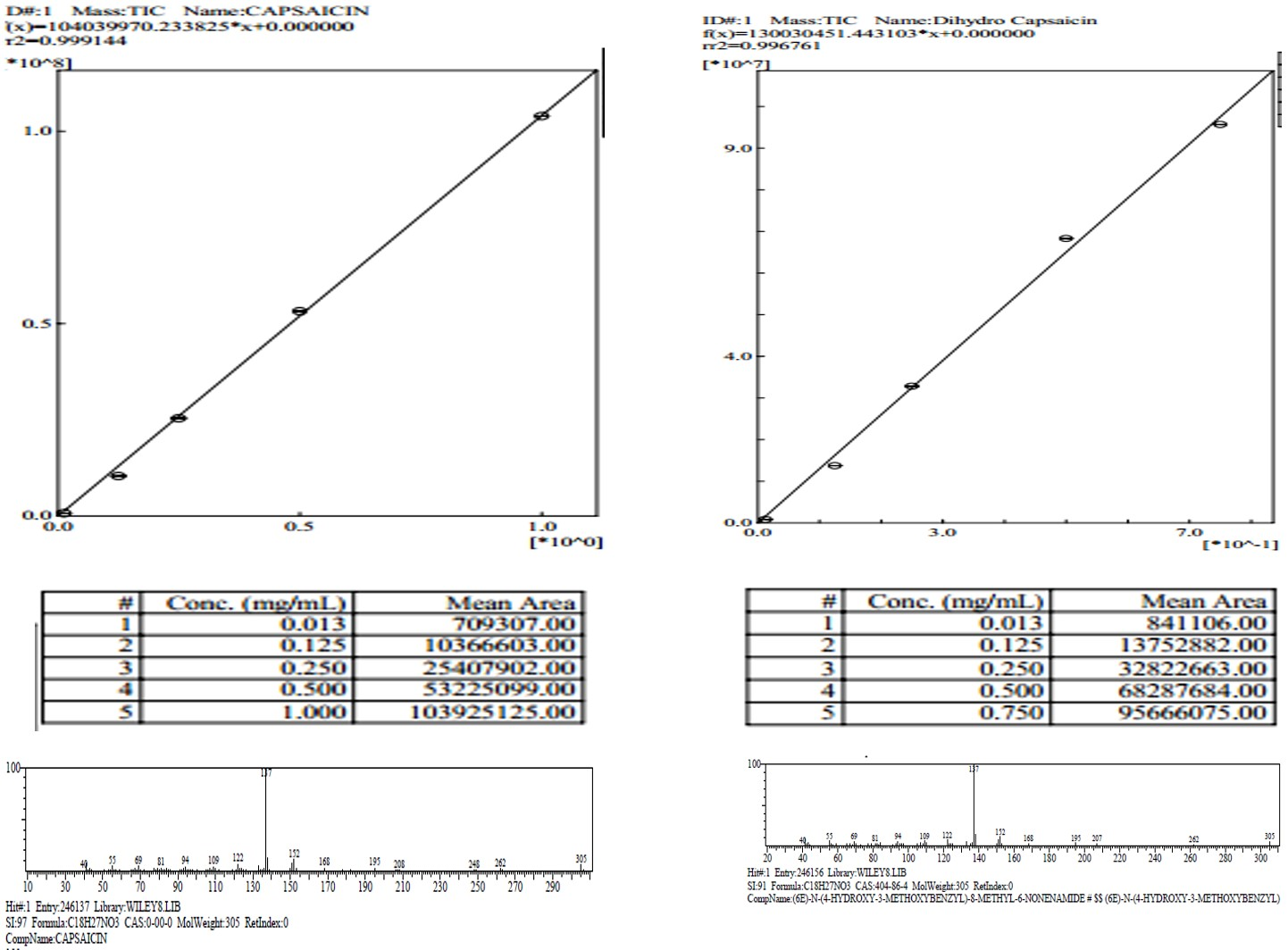

Supplement: S1 Fig — (TIF) [file pone.0167791.s001.tif]

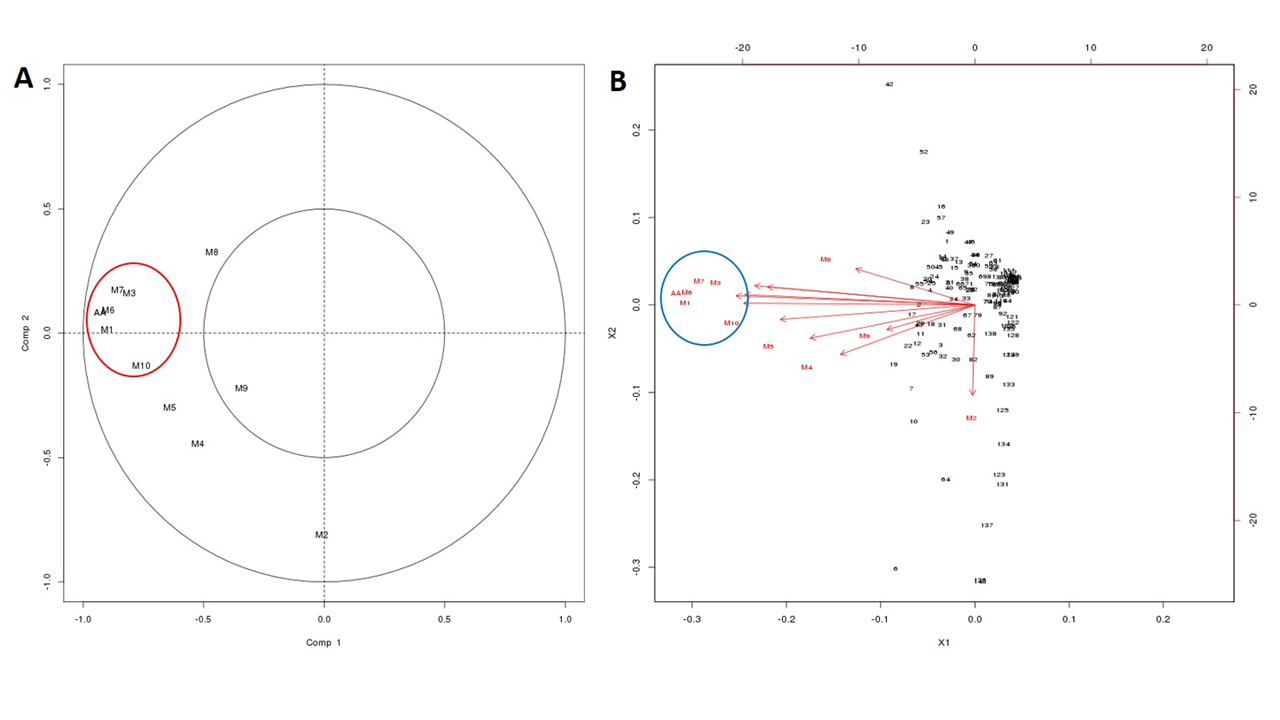

Supplement: S2 Fig — (A) Correlation circle plot shows that there is a similar correlation pattern between certain metabolites and antioxidant activity in majority of the Capsicum species from (C. annuum, C. frutescens and C. chinense). These metabolites are identified and represented as M1 (hexadecanoic or palmitic acid), M3 cyclopentane, M6 (capsaicin), M7 dihydrocapsaicin, M10 (α-tocopherol or vitamin E) and AA (antioxidant activity). The strongly correlated metabolites were projected in the same direction from the origin of the circle. The distance from the origin indicates the strong association of the metabolites. (B) Biplot analysis showing the association between the Capsicum accessions and metabolites. Majority of the accessions from the three species of Capsicum exhibited similar pattern of metabolites correlation. The angle between the arrows (vectors) showed inversely proportional to the correlation of metabolites. Highly correlated metabolites point in the same direction; uncorrelated metabolites are at right angles to each other. (TIF) [file pone.0167791.s002.tif]

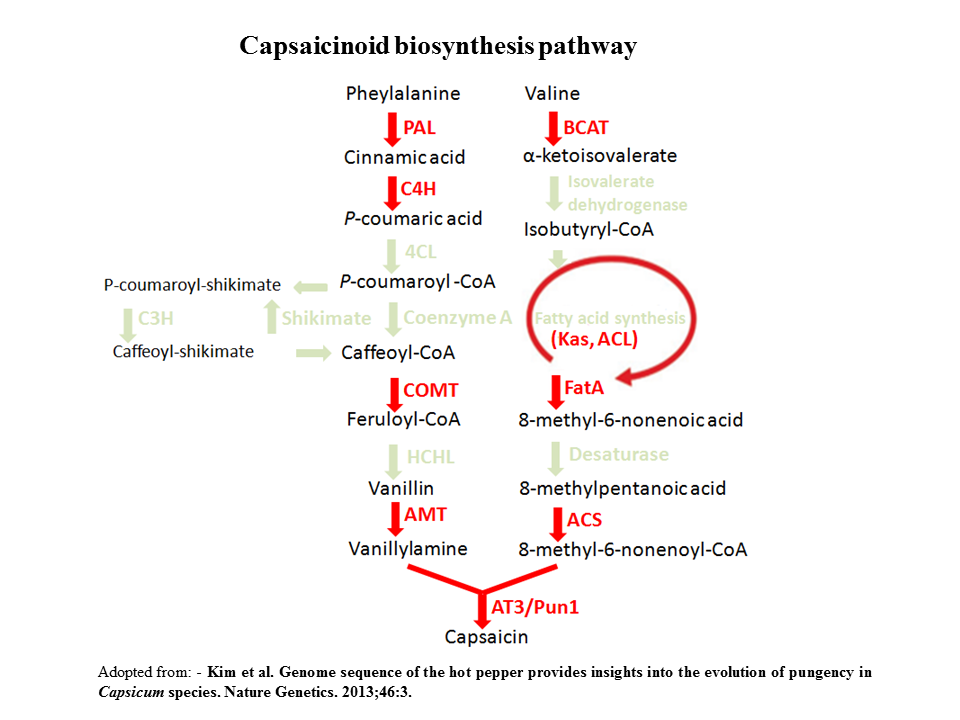

Supplement: S3 Fig — (TIF) [file pone.0167791.s003.tif]
