# Supplementary material for: Comparative Analysis of Fruit Metabolites and Pungency Candidate Genes Expression between Bhut Jolokia and Other Capsicum Species
Source: PLoS One. 2016 Dec 9;11(12):e0167791. doi: 10.1371/journal.pone.0167791 (PMC5147997; doi:10.1371/journal.pone.0167791)
Supplement: S1 Table — (DOCX) [file pone.0167791.s004.docx]

| **Locations** | **Latitude** | **Longitude** |
| --- | --- | --- |
| Guwahati | 26.144517 | 91.736237 |
| Jorhat | 26.746520 | 94.202586 |
| Tinsukia | 27.492191 | 95.346775 |
| Tezpur | 26.652849 | 92.792559 |
| Silchar | 24.833271 | 92.778905 |
| Nagaon | 26.346371 | 92.684043 |
| kokrajhar | 26.401436 | 90.266699 |
| Peren | 47.611160 | 17.804975 |
| Dimapur | 25.862989 | 93.753666 |
| Kohima | 25.658596 | 94.105331 |
| Bishnupur | 24.630360 | 93.759829 |
| Ukhrul | 25.095386 | 94.361652 |
| Imphal | 24.817011 | 93.936844 |
| Champhai | 23.456571 | 93.328193 |
| Lunglei | 22.867069 | 92.765536 |
| Nongstoin | 25.521309 | 91.255411 |
| Shillong | 25.578773 | 91.893254 |
| Baghmara | 25.193491 | 90.634631 |
| Kozhikode | 11.258753 | 75.780410 |
| Ernakulam | 9.981636 | 76.299884 |
| Udhampur | 32.915985 | 75.141617 |
| Jammu | 32.726602 | 74.857026 |
| New Delhi | 28.613939 | 77.209021 |

S 1 Table. Geographical locations and coordinates of sampling sites.
