# Supplementary material for: Comparative Analysis of Fruit Metabolites and Pungency Candidate Genes Expression between Bhut Jolokia and Other Capsicum Species
Source: PLoS One. 2016 Dec 9;11(12):e0167791. doi: 10.1371/journal.pone.0167791 (PMC5147997; doi:10.1371/journal.pone.0167791)
Supplement: S3 Table — (DOCX) [file pone.0167791.s006.docx]

**S 3 Table. Concentration of different capsaicinoid components (in µg/g DW^a^ of fruit) and antioxidant activities observed in *Capsicum* accessions**.

| Accessions | Specie*s* | Capsaicin in µg | Dihydro  capsaicin in µg | Nordihydro  capsaicin in µg | Nonivamide in µg | Capsaicinoids in µg | SHU | Antioxidant activity (%) |
| --- | --- | --- | --- | --- | --- | --- | --- | --- |
| ACC 1 | *C. chinense* | 22700 ± 1017 | 16017.141 ± 875 | 190.3 ± 19 | 163.2 ± 7.8 | 39070.6 ± 1918.8 | 656058.3 ± 30708 | 64.67 ± 4.163 |
| ACC 2 | *C. chinense* | 35661 ± 483 | 18961 ± 621 | 82.8 ± 2.5 | 21.3 ± 1.2 | 54726.1 ± 1107.7 | 889074.2 ± 17808 | 77.83 ± 4.335 |
| ACC 3 | *C. chinense* | 17562.8 ± 1227 | 12125.18 ± 989 | 61.28 ± 8.7 | 0.3898 ± 0.12 | 29749.7 ± 2224.8 | 483711.4 ± 35759 | 63.18 ± 2.600 |
| ACC 4 | *C. chinense* | 33690 ± 1910 | 15507.760 ± 1297 | 30.4 ± 1.9 | 15.2 ± 2.7 | 49243.4 ± 3211.6 | 796309.5 ± 51675 | 74.57 ± 1.791 |
| ACC 5 | *C. chinense* | 20245.37 ± 1580 | 12202.21 ± 1109 | 1.03 ± 0.09 | 15.1 ± 4.4 | 32463.7 ± 2693.5 | 523890.6 ± 43334 | 66.28 ± 3.256 |
| ACC 6 | *C. chinense* | 39160 ± 819 | 15995 ± 797 | 39.6 ± 2.6 | 22.6 ± 3.6 | 55217.2 ± 1622.2 | 893757.5 ± 26074 | 77.56 ± 2.542 |
| ACC 7 | *C. chinense* | 39460 ± 1429 | 16260 ± 510 | 35.9 ± 1.7 | 33 ± 3.7 | 55788.9 ± 1944.4 | 903466.7 ± 31267 | 81.22 ± 0.813 |
| ACC 8 | *C. chinense* | 42797 ± 1170 | 20663 ± 770 | 37.5 ± 16.3 | nd^b^ | 63497.5 ± 1956.3 | 1025193 ± 31385 | 80.65 ± 2.000 |
| ACC 9 | *C. chinense* | 20342.97 ± 1819 | 12696.04 ± 393 | 2.2 ± 0.5 | nd | 33041.2 ± 2212.5 | 532132.6 ± 35617 | 51.00 ± 1.732 |
| ACC 10 | *C. chinense* | 28780 ± 1228 | 16256 ± 956 | 90.3 ± 11 | 816.7 ± 18.4 | 45943.0 ± 2213.4 | 808613.9 ± 35433 | 81.78 ± 1.955 |
| ACC 11 | *C. chinense* | 38840.39 ± 2189 | 19211.299 ± 1223 | 75.1 ± 7.5 | 54 ± 3.1 | 58180.8 ± 3422.6 | 946584.5 ± 55031 | 76.51 ± 2.307 |
| ACC 12 | *C. chinense* | 31340 ± 1703 | 15172.189 ± 941 | 67.1 ± 4.7 | 76.4 ± 4.9 | 46655.7 ± 2653.6 | 762115.3 ± 42657 | 78.68 ± 2.477 |
| ACC 13 | *C. chinense* | 19582.043 ± 583 | 14046.84 ± 927 | 82.4 ± 1.6 | nd | 33711.3 ± 1511.6 | 549088.2 ± 24325 | 57.66 ± 3.161 |
| ACC 14 | *C. chinense* | 25180.56 ± 3214 | 15581.82 ± 1643 | 108.9 ± 12 | 8.6 ± 0.8 | 40879.9 ± 4869.8 | 667193.2 ± 78316 | 79.96 ± 0.654 |
| ACC 15 | *C. chinense* | 25300 ± 1874 | 14095.53 ± 917 | 14 ± 5 | nd | 39409.5 ± 2796.0 | 635570.0 ± 44981 | 68.14 ± 3.352 |
| ACC 16 | *C. chinense* | 26780 ± 918 | 15395.73 ± 887 | 432.2 ± 73 | 211.1 ± 9.2 | 42819.0 ± 1887.2 | 738645.0 ± 29824 | 54.80 ± 2.211 |
| ACC 17 | *C. chinense* | 25973.298 ± 1178 | 15789.12 ± 1290 | 357.6 ± 71.6 | 252.2 ± 21.9 | 42372.2 ± 2561.5 | 728834.1 ± 40602 | 69.14 ± 2.242 |
| ACC 18 | *C. chinense* | 34340 ± 2010 | 19810 ± 1271 | 81.7 ± 20.7 | 39.4 ± 6.4 | 54271.1 ± 3308.1 | 883037.9 ± 53075 | 70.73 ± 0.478 |
| ACC 19 | *C. chinense* | 36308 ± 2173 | 22525 ± 633 | 122.3 ± 10.7 | 17.1 ± 1.3 | 58972.4 ± 2818.0 | 960158.4 ± 45288 | 69.34 ± 1.935 |
| ACC 20 | *C. chinense* | 53900 ± 1012 | 6684 ± 124.0 | 8.63 ± 1.3 | nd | 60592.6 ± 1137.3 | 976204.7 ± 18301 | 78.39 ± 0.905 |
| ACC 21 | *C. chinense* | 22829.66 ± 2813 | 13250.21 ± 1189 | 149.9 ± 8.5 | nd | 36229.8 ± 4010.5 | 594826.6 ± 64511 | 57.51 ± 1.290 |
| ACC 22 | *C. chinense* | 54543 ± 882 | 4682 ± 128 | 21 ± 0.88 | 17.1 ± 1.5 | 59263.1 ± 1012.4 | 957048.7 ± 16282 | 76.06 ± 1.290 |
| ACC 23 | *C. chinense* | 42646 ± 546 | 21720 ± 886 | nd | 11.01 ± 1.1 | 64377.0 ± 1433.1 | 1037305 ± 23065 | 79.75 ± 2.083 |
| ACC 24 | *C. chinense* | 40285.68 ± 751.5 | 16739.37 ± 414 | 4.45 ± 1.2 | 51.223 ± 2 | 57080.7 ± 1168.7 | 923229.7 ± 18794 | 67.77 ± 3.040 |
| ACC 25 | *C. chinense* | 52823.17 ± 634 | 3728.5 ± 212 | 3.014 ± 0.9 | 52.5 ± 1.8 | 56607.2 ± 848.7 | 915592.1 ± 13645 | 77.91 ± 3.643 |
| ACC 26 | *C. chinense* | 31573.47 ± 629 | 5083.07 ± 314 | 0.25 ± 0.02 | 3.6586 ± 0.4 | 36660.4 ± 943.4 | 590529.9 ± 16186 | 61.01 ± 2.388 |
| ACC 27 | *C. chinense* | 12966.83 ± 427 | 4963.51 ± 253 | nd | nd | 17930.3 ± 680.0 | 288678.5 ± 10948 | 43.86 ± 2.066 |
| ACC 28 | *C. chinense* | 31469.84 ± 394 | 7074.9 ± 179 | 0.81 ± 0.25 | 58.97 ± 1.8 | 38604.5 ± 575.1 | 626070.6 ± 9244 | 63.37 ± 0.947 |
| ACC 29 | *C. chinense* | 39015.44 ± 1398 | 18667.468 ± 875 | 62.2 ± 5.5 | 20.1 ± 1.55 | 57765.2 ± 2280.1 | 936328.6 ± 36660 | 71.28 ± 3.552 |
| ACC 30 | *C. chinense* | 28128.86 ± 2889 | 13506.23 ± 1890 | 67.3 ± 23.3 | 40.5 ± 1.6 | 41742.9 ± 4803.9 | 680309.8 ± 77173 | 46.81 ± 0.612 |
| ACC 31 | *C. chinense* | 45597 ± 3682 | 3916 ± 174 | 12.25 ± 3.4 | 64.8 ± 6.9 | 49590.1 ± 3866.3 | 804260.5 ± 62176 | 64.82 ± 1.880 |
| ACC 32 | *C. chinense* | 37639 ± 1135 | 18979 ± 912 | 20.2 ± 4.4 | 26.8 ± 6.1 | 56665.0 ± 2057.5 | 915893.9 ± 33053 | 65.41 ± 1.426 |
| ACC 33 | *C. chinense* | 32779.75 ± 993 | 2567.33 ± 1187 | 17.6 ± 6.2 | nd | 35364.7 ± 2186.2 | 570724.8 ± 35155 | 57.71 ± 1.368 |
| ACC 34 | *C. chinense* | 45397 ± 2786 | 3994 ± 829 | 14.2 ± 2.1 | 10.8 ± 1.7 | 49416.0 ± 3618.8 | 797509.3 ± 58236 | 68.03 ± 2.025 |
| ACC 35 | *C. chinense* | 21005.62 ± 2369 | 11083.36 ± 1257 | 3.12 ± 0.1 | 0.302 ± 0.089 | 32092.4 ± 3626.2 | 516950.9 ± 58388 | 48.13 ± 0.441 |
| ACC 36 | *C. chinense* | 21659 ± 1214 | 10058.028 ± 978 | 1.16 ± 0.8 | 0.8376 ± 0.22 | 31719.0 ± 2193.0 | 510829.5 ± 35300 | 46.64 ± 0.528 |
| ACC 37 | *C. chinense* | 36550.543 ± 1632 | 10456.827 ± 1132 | 33.1 ± 1.6 | 13.9 ± 0.9 | 47054.4 ± 2766.5 | 761175.7 ± 44523 | 66.34 ± 1.900 |
| ACC 38 | *C. chinense* | 24102.85 ± 2766 | 16430.677 ± 2104 | 20.7 ± 1.1 | 14.8 ± 1.7 | 40569.0 ± 4872.8 | 655876.4 ± 78432 | 65.73 ± 2.318 |
| ACC 39 | *C. chinense* | 13716.82 ± 1928 | 11868.5 ± 1102 | 184.2 ± 20.2 | 232 ± 4.4 | 26001.5 ± 3054.6 | 450398.2 ± 49011 | 55.24 ± 2.395 |
| ACC 40 | *C. chinense* | 35784 ± 267 | 14444 ± 289 | 31.2 ± 1.3 | 26.4 ± 0.9 | 50285.6 ± 558.2 | 814001.1 ± 8971 | 66.69 ± 0.629 |
| ACC 41 | *C. chinense* | 35967 ± 334 | 15585 ± 715 | 8.2 ± 1.9 | 12.5 ± 5.1 | 51572.7 ± 1056.0 | 831899.7 ± 16953 | 68.72 ± 2.785 |
| ACC 42 | *C. chinense* | 34606 ± 909.1 | 22764 ± 606 | 914.1 ± 14.1 | 43.5 ± 3.4 | 58327.6 ± 1532.6 | 1012670 ± 24555 | 78.51 ± 0.985 |
| ACC 43 | *C. chinense* | 45474.44 ± 1952 | 11545.21 ± 1002 | 12.7 ± 2.5 | 17.47 ± 4.65 | 57049.8 ± 2961.2 | 920804.6 ± 47625 | 74.05 ± 1.371 |
| ACC 44 | *C. chinense* | 17907.47 ± 3238 | 19202.16 ± 1192 | 27.3 ± 6.1 | 17.9 ± 1.5 | 37154.8 ± 4437.6 | 601650.6 ± 71393 | 60.38 ± 2.247 |
| ACC 45 | *C. chinense* | 47893 ± 2734 | 13991 ± 3030 | 17.9 ± 5.2 | 18.8 ± 2.9 | 61920.7 ± 5772.1 | 999726.6 ± 92875 | 72.45 ± 1.137 |
| ACC 46 | *C. chinense* | 26562.133 ± 699 | 13487.32 ± 125 | 62.611 ± 1.3 | 6.678 ± 0.7 | 40118.7 ± 826.0 | 651233.3 ± 13284 | 45.01 ± 3.504 |
| ACC 47 | *C. chinense* | 30030.28 ± 388 | 12227.92 ± 436 | 68.557 ± 1.6 | 10.5812 ± 1.9 | 42337.3 ± 827.5 | 687706.2 ± 13298 | 46.49 ± 3.342 |
| ACC 48 | *C. chinense* | 45457.1898 ± 851 | 11368.761 ± 227 | 99.3783 ± 4.2 | 42.8476 ± 1.1 | 56968.2 ± 1083.3 | 928081.9 ± 17404 | 69.49 ± 3.342 |
| ACC 49 | *C. chinense* | 41254 ± 541 | 13627 ± 608 | 96.92 ± 8.3 | 17.51 ± 0.9 | 54995.4 ± 1158.2 | 894208.5 ± 18584 | 55.81 ± 2.454 |
| ACC 50 | *C. chinense* | 42593.35 ± 1506 | 15090.934 ± 978 | 107.64 ± 43.3 | 27.8395 ± 3.4 | 57819.8 ± 2530.7 | 941289.0 ± 40426 | 67.57 ± 0.563 |
| ACC 51 | *C. chinense* | 30103 ± 275 | 9275 ± 94 | 74.6 ± 2.5 | 11.666 ± 2.4 | 39464.3 ± 373.9 | 641996.8 ± 5986 | 47.54 ± 0.355 |
| ACC 52 | *C. chinense* | 23741.785 ± 2887 | 14135.496 ± 1659 | 669.6 ± 99.2 | 4.94 ± 1.4 | 38551.8 ± 4646.6 | 672551.4 ± 74126 | 63.19 ± 2.714 |
| ACC 53 | *C. chinense* | 32564 ± 1036 | 10149 ± 1710 | 243.1 ± 25.8 | 741.5 ± 17.2 | 43697.6 ± 2789.0 | 778505.5 ± 44608 | 63.27 ± 1.260 |
| ACC 54 | *C. chinense* | 29753.99 ± 2018 | 26455.189 ± 779 | 25.266 ± 1.2 | 231.16 ± 22.8 | 56465.6 ± 2821.0 | 928583.8 ± 45252 | 75.68 ± 1.498 |
| ACC 55 | *C. chinense* | 28210.767 ± 3129 | 13897.054 ± 1039 | 60.196 ± 12 | 601.33 ± 11.3 | 42769.3 ±4191.3 | 738856.0 ± 67320 | 60.23 ± 7.333 |
| ACC 56 | *C. chinense* | 29481 ± 576 | 22520 ± 448 | 242.8 ± 19.8 | 5.665 ± 1.9 | 52249.5 ± 1045.7 | 860317.5 ± 16688 | 67.75 ± 2.954 |
| ACC 57 | *C. chinense* | 17133.51 ± 661 | 2605.24 ± 534 | 4.27 ± 0.4 | 16.7 ± 3.6 | 19759.7 ± 1199.0 | 319727.4 ± 19276 | 47 ± 2.167 |
| ACC 58 | *C. chinense* | 11528.33 ± 768 | 11177.65 ± 439 | nd | nd | 22706.0 ± 1207.0 | 365566.2 ± 19432 | 41.19 ± 3.379 |
| ACC 59 | *C. chinense* | 25816.897 ± 4287 | 9858.895 ± 937 | 105.99 ± 43.2 | 101.31 ± 2.9 | 35883.1 ±5270.1 | 593557.7 ± 84534 | 54.57±2.963 |
| ACC 60 | *C. chinense* | 12233.567 ± 1314 | 4656.38 ± 1092 | nd | 10.533 ± 2.5 | 16900.5 ± 2408.5 | 272897.1 ± 38759 | 40.69±2.138 |
| ACC 61 | *C. chinense* | 15573 ± 3913 | 6970.59 ± 1374 | 89.32 ± 10.9 | 20.67 ± 1.7 | 22653.6 ± 5299.6 | 373160.1 ± 85237 | 48.12±7.957 |
| ACC 62 | *C. chinense* | 15748 ± 3211 | 3386.6 ± 1091 | nd | nd | 19134.6 ± 4302.0 | 308067.0 ± 69262 | 44.77±0.8269 |
| ACC 63 | *C. chinense* | 28744.3 ± 392 | 3278.03 ± 812 | 33.145 ± 5.7 | nd | 32055.5 ± 1209.7 | 518642.0 ± 19437 | 56.8 ± 0.9792 |
| ACC 64 | *C. frutescens* | 16156.64 ± 1431 | 13824.18 ± 1247 | 48.8 ± 11.3 | 2.2591 ± 1.2 | 30031.9 ± 2690.5 | 487437.3 ± 43231 | 43.55 ± 3.211 |
| ACC 65 | *C. frutescens* | 17546.22 ± 1671 | 12728.82 ± 1332 | 1.261 ± 0.7 | 0.81 ± 0.5 | 30277.1 ± 3004.2 | 487619.6 ± 48359 | 47.40 ± 2.114 |
| ACC 66 | *C. frutescens* | 14354.11 ± 678 | 11538.43 ± 591 | 81 ± 3.1 | 5.503 ± 1.5 | 25979.0 ± 1273.6 | 424909.1 ± 20473 | 47.19 ± 1.054 |
| ACC 67 | *C. frutescens* | 14102.06 ± 2039 | 13260 ± 1821 | 70.6 ± 8.9 | 20.3 ± 0.6 | 27453.0 ± 3869.5 | 448962.5 ± 62234 | 44.32 ± 1.173 |
| ACC 68 | *C. frutescens* | 13612.98 ± 1008 | 9482.56 ± 1103 | 15.1 ± 5.2 | 0.487 ± 0.2 | 23111.1 ± 2116.4 | 373287.2 ± 34037 | 40.91 ± 1.894 |
| ACC 69 | *C. frutescens* | 13880.61 ± 1788 | 9750 ± 1290 | 14.2 ± 3.3 | 1.102 ± 0.4 | 23645.9 ± 3081.7 | 381874.7 ± 49590 | 39.21 ± 1.581 |
| ACC 70 | *C. frutescens* | 14435.97 ± 1671 | 12332.22 ± 1025 | 30.2 ± 7.2 | 2.669 ± 0.4 | 26801.1 ± 2703.6 | 434021.9 ± 43476 | 34.36 ± 3.735 |
| ACC 71 | *C. frutescens* | 5075.004 ± 1067 | 1719.371 ± 135 | 0.72 ± 0.3 | 0.5601 ± 0.25 | 6795.7 ± 1202.6 | 109508.0 ± 19357 | 33.94 ± 3.331 |
| ACC 72 | *C. frutescens* | 11502.05 ± 884 | 1722.95 ± 1149 | 73.48 ± 18.8 | 0.051 ± 0.009 | 13298.5 ± 2051.8 | 219761.2 ± 32906 | 41.94 ± 3.824 |
| ACC 73 | *C. frutescens* | 12336.77 ± 2211 | 5011.66 ± 791 | 3.123 ± 0.75 | 0.35 ± 0.187 | 17351.9 ± 3002.9 | 279632.3 ± 48340 | 48.00 ± 4.696 |
| ACC 74 | *C. frutescens* | 5816.54 ± 498 | 2082.31 ± 207 | 10.8 ± 0.6 | 0.6884 ± 0.24 | 7910.3 ± 705.8 | 128239.2 ± 11358 | 34.21 ± 7.138 |
| ACC 75 | *C. frutescens* | 9440.67 ± 762 | 3118.53 ± 392 | 0.035 ± 0.008 | 0.327 ± 0.14 | 12559.6 ± 1154.1 | 202236.4 ± 18580 | 36.31 ± 3.160 |
| ACC 76 | *C. frutescens* | 12310.41 ± 1489 | 4240.36 + 1023 | 0.3362 ± 0.22 | nd | 16551.1 ± 2512.2 | 266498.6 ± 40445 | 43.83 ± 3.620 |
| ACC 77 | *C. frutescens* | 15218.56 ± 1300 | 3411.92 ± 372 | 1.197 ± 0.6 | 41.422 ± 8 | 18673.1 ± 1680.6 | 303872.8 ± 26998 | 49.73 ± 3.952 |
| ACC 78 | *C. frutescens* | 5028.8 ± 1217 | 388.4 ± 21 | 83.9 ± 1.9 | 0.461 ± 0.211 | 5501.6 ± 1240.1 | 95062.0 ± 19951 | 31.95±3.619 |
| ACC 79 | *C. frutescens* | 7388.7 ± 758 | 3866.3 ± 591 | 15.43 ± 0.9 | 113.41 ± 1.1 | 11383.8 ± 1351.0 | 193074.2 ± 21737 | 36.98±9.029 |
| ACC 80 | *C. frutescens* | 11503.45 ± 178 | 3247.43 ± 116 | 21.6 ± 1.2 | 85.809 ± 0.9 | 14858.3 ± 296.1 | 247392.4 ± 4752 | 39.37±6.095 |
| ACC 81 | *C. annuum* | 5190.86 ± 139 | 1028.24 ± 47 | 1.881 ± 0.9 | 0.116 ± 0.089 | 6221.1 ± 187.0 | 100313.0 ± 3003 | 29.66 ± 4.326 |
| ACC 82 | *C. annuum* | 4990 ± 110 | 1132.89 ± 102 | 13.8 ± 0.7 | 0.15 ± 0.038 | 6136.8 ± 212.7 | 99875.5 ± 3420 | 35.38 ± 2.987 |
| ACC 83 | *C. annuum* | 1459.019 ± 294 | 577.64 ± 116 | nd | nd | 2036.7 ± 410.0 | 32790.2 ± 6601 | 27.07 ± 3.201 |
| ACC 84 | *C. annuum* | 3983.87 ± 428 | 3227.13 ± 471 | 0.196 ± 0.08 | 0.021 ± 0.008 | 7211.2 ± 899.1 | 116117.3 ± 14474 | 33.20 ± 4.947 |
| ACC 85 | *C. annuum* | 1986.65 ± 866 | 3651.31 ± 173 | 5.812 ± 1.3 | 11.3 ± 3.4 | 5655.1 ± 1043.7 | 92351.2 ± 16771 | 28.48 ± 1.590 |
| ACC 86 | *C. annuum* | 1024.57 ± 34 | 524.1 ± 9 | nd | nd | 1548.7 ± 43 | 24933.6 ± 692 | 17.24 ± 1.978 |
| ACC 87 | *C. annuum* | nd | nd | nd | nd | nd | nd | 5.67 ± 9.815 |
| ACC 88 | *C. annuum* | 1030.67 ± 51 | 512.79 ± 23 | 0.733 ± 0.4 | 2.146 ± 0.5 | 1546.3 ± 74.9 | 25115.3 ± 1199 | 15.06 ± 3.398 |
| ACC 89 | *C. annuum* | 1793.27 ± 119 | 459.37 ± 67 | 0.691 ± 0.52 | 0.422 ± 0.3 | 2253.8 ± 186.8 | 36370.5 ± 3002 | 22.23 ± 0.44 |
| ACC 90 | *C. annuum* | 3774.6 ± 15 | 104.28 ± 8 | nd | nd | 3878.9 ± 23 | 62449.9 ± 370 | 19.15 ± 0.635 |
| ACC 91 | *C. annuum* | 330.84 ± 19.38 | 151.26 ± 67.98 | 0.118 ± 0.076 | 8.64 ± 0.7 | 490.9 ± 88.1 | 8567.4 ± 1413 | 9.33 ± 0.7430 |
| ACC 92 | *C. annuum* | 1099.65 ± 56 | 67.18 ± 25 | 2.11 ± 0.9 | 9.332 ± 0.5 | 1178.3 ± 82.4 | 19840.8 ± 1317 | 5.378 ± 2.301 |
| ACC 93 | *C. annuum* | 335.57 ± 15.5 | 103.46 ± 11 | 0.58 ± 0.33 | 0.04 ± 0.007 | 439.65 ± 26.83 | 7125.7 ± 429 | 18.05 ± 0.582 |
| ACC 94 | *C. annuum* | 655.61 ± 47 | 328.1 ± 33 | 0.141 ± 0.1 | 0.21 ± 0.11 | 984.1 ± 80.2 | 15869.8 ± 1289 | 17.23 ±1.546 |
| ACC 95 | *C. annuum* | 101.18 ± 9.87 | 61.11 ± 3.56 | nd | 6.26 ± 0.43 | 168.5 ± 13.9 | 3188.4 ± 220 | 2 8.16 ± 4.55 |
| ACC 96 | *C. annuum* | 268.76 ± 41 | 156.22 ± 13.9 | 0.0874 ± 0.04 | 0.06 ± 0.03 | 425.1 ± 55.0 | 6855.4 ± 884 | 5.381 ± 9.364 |
| ACC 97 | *C. annuum* | 233.52 ± 17 | 92.7 ± 4.85 | 0.0994 ± 0.06 | 0.02 ± 0.008 | 326.3 ± 21.9 | 5262.8 ± 352 | 4.599 ±13.4 |
| ACC 98 | *C. annuum* | 176.1 ± 24 | 97.6 ± 13 | 0.0935 ± 0.06 | 0.02 ± 0.005 | 273.8 ± 37.1 | 4416.8 ± 596 | 3.52 ± 0.0088 |
| ACC 99 | *C. annuum* | 233.5 ± 39 | 171.9 ± 20 | 0.141 ± 0.073 | nd | 405.5 ± 59.1 | 6540.0 ± 950 | 11.05 ± 2.485 |
| ACC 100 | *C. annuum* | 1248.23 ± 319 | 365.1 ± 87 | 0.3321 ± 0.13 | 0.013 ± 0.009 | 1613.7 ± 406.1 | 26006.6 ± 6537 | 24.3 ± 2.471 |
| ACC 101 | *C. annuum* | 719.7 ± 60 | 251 ± 32 | 0.2403 ± 0.09 | 3.91 ± 0.388 | 974.9 ± 92.5 | 16010.3 ± 1485 | 12.8 ± 4.336 |
| ACC 102 | *C. annuum* | 2243.98 ± 222 | 413.3 ± 38 | 0.308 ± 0.14 | 0.26 ± 0.15 | 2657.8 ± 260.3 | 42834.7 ± 4188 | 22.51 ± 8.73 |
| ACC 103 | *C. annuum* | 1063.12 ± 109 | 348.87 ± 15 | 0.3842 ± 0.17 | nd | 1412.4 ± 124.2 | 22768.8 ± 1997 | 11.6 ± 4.036 |
| ACC 104 | *C. annuum* | 985.33 ± 50 | 534.23 ± 11 | 8.9 ± 0.3 | 0.019 ± 0.009 | 1528.5 ± 61.3 | 25294.3 ± 984 | 12.88 ± 5.995 |
| ACC 105 | *C. annuum* | 2056.367 ± 717 | 581.09 ± 65 | 2.598 ± 0.7 | nd | 2640.1 ± 782.7 | 42704.7 ± 12596 | 21.73 ± 2.942 |
| ACC 106 | *C. annuum* | 1498.01 ± 189 | 958.729 ± 62 | 10.24 ± 0.5 | 0.017 ± 0.007 | 2467.0 ± 251.5 | 40506.9 ± 4045 | 19.21 ± 1.544 |
| ACC 107 | *C. annuum* | 1287.38 ± 253 | 982.2 ± 59 | 2.43 ± 0.7 | 0.024 ± 0.004 | 2272.0 ± 312.7 | 36768.4 ± 5029 | 20.26 ± 2.826 |
| ACC 108 | *C. annuum* | 1486.76 ± 284 | 774.9 ± 60 | 6.32 ± 0.7 | 0.012 ± 0.007 | 2268.0 ± 344.7 | 37001.6 ± 5544 | 26.04 ± 13.94 |
| ACC 109 | *C. annuum* | 1659.98 ± 192 | 745.1 ± 51 | 0.224 ± 0.095 | 4.27 ± 1 | 2409.6 ± 244.1 | 39135.4 ± 3922 | 23.74 ± 2.814 |
| ACC 110 | *C. annuum* | 4256 ± 719 | 794.56 ± 38 | 16.223 ± 2.1 | 3.211 ± 0.5 | 5070.0 ± 759.6 | 83118.2 ± 12211 | 31.32 ± 6.15 |
| ACC 111 | *C. annuum* | 2875.09 ± 193 | 1022.64 ± 73 | 7.922 ± 0.9 | 0.1015 ± 0.06 | 3905.8 ± 267.0 | 63499.5 ± 4291 | 27.73 ± 2.004 |
| ACC 112 | *C. annuum* | 1537.47 ± 371 | 869.66 ± 109 | 7.256 ± 0.4 | nd | 2414.4 ± 480.4 | 39429.6 ± 7731 | 17.69 ± 2.005 |
| ACC 113 | *C. annuum* | 1204.43 ± 234 | 648.98 ± 18 | 2.742 ± 0.8 | 0.028 ± 0.012 | 1856.2 ± 252.8 | 30097.5 ± 4064 | 13.86 ± 2.601 |
| ACC 114 | *C. annuum* | 873.71 ± 77 | 437.58 ± 43 | 4.34 ± 0.7 | 0.006 ± 0.004 | 1315.6 ± 120.7 | 21516.0 ± 1938 | 10.67 ± 3.561 |
| ACC 115 | *C. annuum* | 1915.087 ± 831 | 812.37 ± 75 | 6.28 ± 1.2 | 0.009 ± 0.003 | 2733.7 ± 907.2 | 44496.9 ± 14597 | 18.2 ± 3.708 |
| ACC 116 | *C. annuum* | 197.2 ± 13 | 25.41 ± 7 | nd | nd | 222.6 ± 20.0 | 3584.0 ± 1 ± 322 | 6.15 ± 5.309 |
| ACC 117 | *C. annuum* | 728.2 ± 78 | 429.7 ± 21 | 9.751 ± 3 | 0.371 ± 0.21 | 1168.0 ± 102.2 | 19583.2 ± 1623 | 9.169 ± 1.471 |
| ACC 118 | *C. annuum* | 1793 ± 117 | 188.9 ± 45 | 1.84 ± 0.48 | 0.202 ± 0.09 | 1983.9 ± 162.6 | 32098.0 ± 2613 | 7.592 ± 4.579 |
| ACC 119 | *C. annuum* | 1282.5 +114 | 451.1 ± 8 | 3.044 ± 1.7 | 0.152 ± 0.075 | 1736.8 ± 123.8 | 28208.0 ± 1980 | 10.34 ± 7.18 |
| ACC 120 | *C. annuum* | 763.2 ± 65 | 223.1 ± 22 | 1.041 ± 0.35 | nd | 987.3 ± 87.4 | 15976.2 ± 1403 | 7.712 ± 11.39 |
| ACC 121 | *C. annuum* | 3666.9 ± 127 | 1258.5 ± 109 | 87.98 ± 2.1 | nd | 5013.4 ± 238.1 | 87481.1 ± 3819 | 17.94 ± 4.576 |
| ACC 122 | *C. annuum* | 549.2 ± 17 | 348.9 ± 12 | 0.41 ± 0.21 | 0.57 ± 0.35 | 899.1 ± 29.6 | 14549.7 ± 472 | 7.143 ± 5.613 |
| ACC 123 | *C. annuum* | 595.8 ± 42 | 146.6 ± 9 | 0.8963 ± 0.43 | 0.25 ± 0.17 | 743.5 ± 51.6 | 12058.5 ± 826 | 9.356 ± 2.668 |
| ACC 124 | *C. annuum* | 756 ± 57 | 55.8 ± 6 | 1.963 ± 1.1 | 0.22 ± 0.095 | 814.0 ± 64.2 | 13273.0 ± 1025 | 8.557 ± 9.218 |
| ACC 125 | *C. annuum* | 1098.5 ± 67 | 83.8 ± 8 | 1.644 ± 0.7 | 0.398 ± 0.15 | 1184.3 ± 75.9 | 19224.5 ± 1215 | 8.89 ± 8.668 |
| ACC 126 | *C. annuum* | 167.3 ± 11 | 25.4 ± 3 | 0.9997 ± 0.58 | 0.231 ± 0.075 | 193.9 ± 14.7 | 3216.7 ± 231 | 9.878 ± 6.11 |
| ACC 127 | *C. annuum* | 2755.7 ± 82 | 1762 ± 23 | 9.24 ± 2.7 | 108.5 ± 0.5 | 4635.4 ± 108.2 | 83576.0 ± 1720 | 27.95 ± 6.695 |
| ACC 128 | *C. annuum* | 2144 ± 91 | 1456 ± 57 | 3.88 ± 0.4 | 0.79 ± 0.3 | 3604.7 ± 148.7 | 58393.1 ± 2389 | 25.96 ± 7.63 |
| ACC 129 | *C. annuum* | 1307.7 ± 66 | 520.9 ± 19 | 1.137 ± 0.6 | 22.6 ± 1.1 | 1852.3 ± 86.7 | 31625.4 ± 1384 | 21.49 ± 3.348 |
| ACC 130 | *C. annuum* | 3268.1 ± 172 | 1941 ± 97 | 83.09 ± 1.5 | 0.12 ± 0.088 | 5292.3 ± 270.6 | 91605.1 ± 4345 | 27.27 ± 3.933 |
| ACC 131 | *C. annuum* | 3089 ± 88 | 826.5 ± 23 | 0.771 ± 0.33 | 20.09 ± 0.7 | 3936.4 ± 112.0 | 64959.5 ± 1796 | 17.7 ± 12.28 |
| ACC 132 | *C. annuum* | 8054.03 ± 872 | 3589 ± 128 | 2.87 ± 1.7 | 174.05 ± 1.4 | 11819.9 ± 1003.1 | 203732.0 ± 16128 | 38.32 ± 6.15 |
| ACC 133 | *C. annuum* | 8666.12 ± 1004 | 3223.4 ± 546 | 1.63 ± 0.7 | 47.88 ± 0.8 | 11939.0 ± 1551.5 | 195977.6 ± 24968 | 34.81 ± 2.544 |
| ACC 134 | *C. annuum* | 3385.87 ± 81 | 1228.8 ± 98 | 32.65 ± 1.9 | 14.27 ± 3.9 | 4661.6 ± 184.8 | 78645.2 ± 2935 | 29.88 ± 1.371 |
| ACC 135 | *C. annuum* | 3399.3 ± 225 | 1873.1 ± 162 | 47.67 ± 4.5 | 0.198 ± 0.066 | 5320.3 ± 391.6 | 89337.2 ± 6273 | 30.33 ± 1.82 |
| ACC 136 | *C. annuum* | 6512.7 ± 121 | 4021.22 ± 77 | 15.622 ± 1.4 | 168.829 ± 2.6 | 10718.4 ± 202.0 | 186581.2 ± 3224 | 36.36 ± 2.486 |

1. DW: Dry weight
2. nd: represents not detected
