# Supplementary material for: Comparative Analysis of Fruit Metabolites and Pungency Candidate Genes Expression between Bhut Jolokia and Other Capsicum Species
Source: PLoS One. 2016 Dec 9;11(12):e0167791. doi: 10.1371/journal.pone.0167791 (PMC5147997; doi:10.1371/journal.pone.0167791)
Supplement: S5 Table — (DOCX) [file pone.0167791.s008.docx]

**S 5 Table.**

List of primer sequences used for expression studies of pungency candidate genes.

| **Gene name** | **Sequence (5'-3')** | **Number of bases** |
| --- | --- | --- |
| ACL-F | CTGCATCTTCCTTCGCTATCT | 21 |
| ACL-R | CGAGTAGCTGGCTTCATTCT | 20 |
| ACS-F | ACGCCGTGAGATTGTAGATG | 20 |
| ACS-R | CTCCGGATGGAATTTCCTACTT | 22 |
| AMT-F | GCTGTCCTTGTAAGCCAGAA | 20 |
| AMT-R | CAGGGTGTCCGGAATAAGTAAA | 22 |
| BCAT-F | GCAAATTGGTGCAGAGAGAATG | 22 |
| BCAT-R | GGAATCCAGCGCTTGTTAGA | 20 |
| C4H-F | TATCCTAGCGCTGCCAATTC | 20 |
| C4H-R | GACTGAACTGTCCACCTTTCTC | 22 |
| COMT-F | TCTGCTGATGAGGGACAATTC | 21 |
| COMT-R | CTGGAAGAGCAAGAGCAGTAG | 21 |
| FatA-F | GTTTCGTATGAGCCGAGTCTT | 21 |
| FatA-R | CTCCAACCTCGTAACACCTAAC | 22 |
| Kas-F | GGACTAATGGGACCTTGTTACTC | 23 |
| Kas-R | CGTTCCACCTGCTACCATAAT | 21 |
| PAL-F | CAGATTGAGGCTGCTGCTATTA | 22 |
| PAL-R | GGAGATGTTCGGAGAGCATAAC | 22 |
| AT3-F | GGTTCTCTCATTACGCCACAA | 21 |
| AT3-R | CCTGATTCTTCTGCCACCTTAG | 22 |

Actin -F GTCCTCTTCCAACCATCCAT 20

Actin -R TACTTTCTCTCTGGTGGTGC 20
